# High-throughput continuous rotation electron diffraction data acquisition *via* software automation

**DOI:** 10.1107/S1600576718015145

**Published:** 2018-11-22

**Authors:** Magdalena Ola Cichocka, Jonas Ångström, Bin Wang, Xiaodong Zou, Stef Smeets

**Affiliations:** a Department of Materials and Environmental Chemistry, Stockholm University, Stockholm, SE-10691, Sweden

**Keywords:** single-crystal electron diffraction, high throughput, crystal screening, structure analysis

## Abstract

Single-crystal electron diffraction (SCED) is emerging as an effective technique to determine and refine the structures of unknown nano-sized crystals. In this work, the implementation of the continuous rotation electron diffraction (cRED) method for high-throughput data collection is described. This is achieved through dedicated software that controls the transmission electron microscope and the camera. Crystal tracking can be performed by defocusing every *n*th diffraction pattern while the crystal rotates, which addresses the problem of the crystal moving out of view of the selected area aperture during rotation. This has greatly increased the number of successful experiments with larger rotation ranges and turned cRED data collection into a high-throughput method. The experimental parameters are logged, and input files for data processing software are written automatically. This reduces the risk of human error, and makes data collection more reproducible and accessible for novice and irregular users. In addition, it is demonstrated how data from the recently developed serial electron diffraction technique can be used to supplement the cRED data collection by automatic screening for suitable crystals using a deep convolutional neural network that can identify promising crystals through the corresponding diffraction data. The screening routine and cRED data collection are demonstrated using a sample of the zeolite mordenite, and the quality of the cRED data is assessed on the basis of the refined crystal structure.

## Abstract 2

A semi-automated routine for continuous rotation electron diffraction has been developed, enabling high-throughput data collection. Serial electron crystallography combined with a deep convolutional network are used to screen for suitable crystals.

## 1. Introduction   

Over the past decade, several techniques have been developed for collecting single-crystal electron diffraction data (SCED) by rotating the crystal in the electron beam. These have reached a stage where data can now be collected routinely to elucidate the structures of submicrometre-sized crystals of organic and inorganic materials (Mugnaioli & Kolb, 2013 ▸; Yun *et al.*, 2015 ▸). Initially, data were collected with discrete steps of the goniometer (Kolb *et al.*, 2007 ▸; Zhang *et al.*, 2010 ▸; Wan *et al.*, 2013 ▸; Shi *et al.*, 2013 ▸). To achieve improved sampling of reciprocal space between the tilt steps, goniometer rotation can be combined with precession (Vincent & Midgley, 1994 ▸; Mugnaioli *et al.*, 2009 ▸) or many small steps of the beam tilt (Zhang *et al.*, 2010 ▸; Wan *et al.*, 2013 ▸). With the recent introduction of dedicated detectors for electron diffraction with fast readout times (Nederlof *et al.*, 2013 ▸; Hattne *et al.*, 2015 ▸), data can now be collected while the crystal is rotating continuously in the electron beam. The benefit of the continuous rotation method (Arndt & Wonacott, 1977 ▸) is that data collection times are greatly reduced and that all of reciprocal space is sampled, with the exception of a small wedge that is excluded when the detector is being read out. This was demonstrated first on radiation-sensitive materials, such as protein nanocrystals (Nederlof *et al.*, 2013 ▸; Nannenga *et al.*, 2014 ▸; Yonekura *et al.*, 2015 ▸; Clabbers *et al.*, 2017 ▸; Xu *et al.*, 2018 ▸), and more recently on organic and inorganic materials, such as metal oxides and metal–organic frameworks (Gemmi *et al.*, 2015 ▸; van Genderen *et al.*, 2016 ▸; Wang *et al.*, 2017 ▸; Köppen *et al.*, 2018 ▸).

To the best of our knowledge, no dedicated software exists to assist with collection of continuous rotation electron diffraction (cRED) data. Because the cRED method is (1) conceptually and experimentally very simple, (2) time efficient, and (3) able to provide high data quality, especially for radiation-sensitive materials by reducing beam exposure, it has become the method of choice in several laboratories for structure determination. We found that there is a strong need for automation to enable high-throughput collection of cRED data. Heretofore, the acquisition of cRED data required a large number of manual operations, and needed the active presence of an operator to locate crystals and supervise the data collection procedure, which still makes it both demanding and time consuming. The most significant complication during data collection has been crystal drift, which is much more difficult to correct for when the crystal is rotating continuously. We also found there is a demand for a well defined, standardized data processing pipeline to make it easier to access standard X-ray crystallographic software such as *XDS* (Kabsch, 2010 ▸) or *DIALS* (Winter *et al.*, 2018 ▸).

Therefore, we have implemented a new routine in the program *Instamatic* (Smeets *et al.*, 2017 ▸) with the aim of automating data acquisition. *Instamatic* is a multi-functional Python toolbox that can interface with the electron microscope and camera, which gives great flexibility in the design of new experiments. With the implementation, the number of steps required to collect data is greatly reduced, crystal tracking during continuous crystal rotation can be achieved by defocusing the diffraction pattern at regular intervals, and experimental log files and instruction files for data processing software are written automatically. This not only makes the cRED method more accessible to novice or irregular users, but also minimizes the risk of human error, which in turn leads to more reproducible experiments and enables high-throughput data collection. *Instamatic* was initially developed for collecting serial electron diffraction (SerialED) data. The SerialED method combines computer-controlled stage translation with beam shift to automatically collect diffraction data on a large number of crystals (Smeets, Zou & Wan, 2018 ▸). Here, we demonstrate that SerialED data can be used to supplement the cRED data collection by automatic screening for suitable crystals using a deep convolutional neural network that can identify *good* crystals through the corresponding diffraction data. This can make searching for crystals more effective, because the most suitable ones can be identified by the algorithm.

In this paper, we describe the practical implementation of the cRED data collection routine in *Instamatic* and the application of SerialED data in combination with machine learning for crystal screening. We also make an assessment of the quality of the collected data using zeolite mordenite as an example.

## 2. Software implementation   

**Figure 1:**
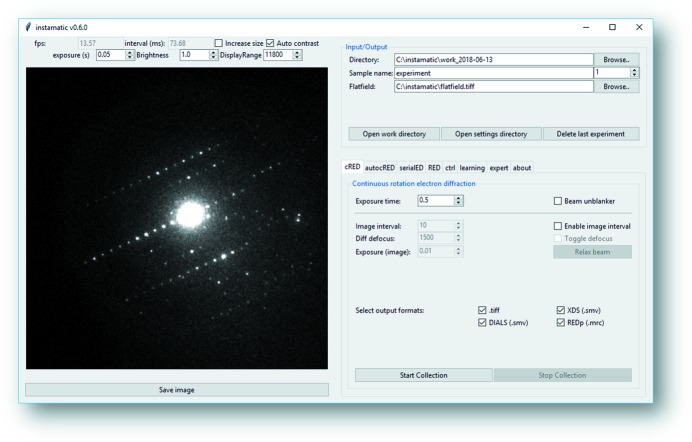
Graphical user interface of the *Instamatic* data collection program.

We have developed the electron diffraction software *Instamatic* (Smeets *et al.*, 2017 ▸), which controls our transmission electron microscope from JEOL and cameras (currently ASI Timepix and Gatan OriusCCD), and implemented routines for crystal screening and cRED data collection. The software is implemented in Python 3.6, which means it has full access to the rich ecosystem of Python libraries and debugging tools. However, the methods described in this paper are presented in a generic manner so that they may be implemented in other software for microscope automation. For microscope control, we have developed an object-oriented wrapper around the TEMCOM application programming interface (API) for control of the JEOL microscope (lenses, deflectors, sample stage *etc*.), which was inspired in part by the *PyScope* library (Suloway *et al.*, 2005 ▸). We have implemented interfaces to the Timepix (ASI) and Gatan OriusCCD cameras using their respective APIs provided by the manufacturers. These interfaces have been abstracted away in a generic microscope class so that other microscopes and cameras may be included in the future. The control interface can be imported into an interactive Python shell (*e.g. IPython*; Perez & Granger, 2007 ▸), which has been very useful for quickly testing new ideas and developing small scripts. On top of the same interface we have developed a modular graphical user interface (GUI) using the *tkinter* library (Tcl/Tk; Fig. 1 ▸). It features a live view of the camera (Timepix), provides an interface for running the different experiments, and offers convenience functions to streamline data I/O and microscope control. Supported formats for data storage are HDF5 (using the *h5py* library; https://www.h5py.org/), SMV and TIFF (both using the implementation in *fabio*; Knudsen *et al.*, 2013 ▸), and MRC (implementation from https://github.com/ezralanglois/arachnid/). The software is developed for Windows, because it needs to access the microscope API.

## 3. Experimental   

The diffraction experiments were performed on a JEOL JEM-2100-LaB_6_ at 200 kV equipped with a 512 × 512 Timepix hybrid pixel detector (55 × 55 µm pixel size, model QTPX-262k) from ASI. Data were collected without the use of a beam stop. A well known zeolite, mordenite (Meier, 1961 ▸), was used as the test sample. The mordenite powder was crushed and dispersed in ethanol, and then subjected to 2 min ultrasonication. One droplet was transferred to a copper grid with continuous carbon film (CF400-Cu-UL from Electron Microscopy Sciences). Excess liquid was removed using filter paper, after which the ethanol was allowed to evaporate. SerialED data were collected using a small condenser aperture (50 µm), spot number 4, and exposure times of 0.1 s for diffraction patterns and 0.5 s for images. Parallel illumination was used in imaging mode. In diffraction mode, the electron beam was focused using the condenser lens (CL1) to give an effective probe diameter of approximately 400 nm. cRED data were collected using a small condenser aperture (70 µm), spot size 2, using parallel illumination with the first (*r*_effective_ = 0.75 µm) and second (*r*_effective_ = 0.35 µm) selected area (SA) apertures. Data were collected using a high-tilt tomography holder (±80°). For the SerialED and cRED experiments, the diffraction patterns were focused to give sharp spots using the intermediate lens (IL1), and the camera length used was 250 mm (giving a maximum resolution *d*_min_ ≃ 0.8 Å).

## 4. Crystal screening using SerialED   

The *Instamatic* software was initially developed to collect SerialED data (Smeets, Zou & Wan, 2018 ▸) and later modified to collect cRED data. In a SerialED experiment, diffraction data are collected from a large number of isolated submicrometre-sized crystals. This is achieved by combining stage translation and beam shift. The sample stage is translated in a raster over a large area (typically several hundreds of micrometres), and at each position of the stage, crystals are detected in imaging mode at low magnification using image-recognition techniques. Once some crystals have been located, the electron beam is focused and shifted to the position of each of the crystals so that a diffraction pattern can be collected. After initial calibrations, the method is fully automated and can survey an area of approximately 400 × 400 µm in an hour. We have shown previously that it is possible to use the electron diffraction data from a large number of crystals for phase analysis (Smeets, Ångström & Olsson, 2018 ▸) and that the merged data are suitable for structure determination (Smeets & Wan, 2017 ▸; Smeets *et al.*, 2018 ▸). In this section, we discuss the application of the SerialED method as a way of screening for *good* crystals for cRED data collection. Because SerialED uses low-magnification images (typically at 2500× magnification) to locate crystals, information about the size, shape and position of crystals is available, but the quality of the data is difficult to judge automatically. For this purpose, we trained a deep convolutional neural network to predict whether a crystal is suitable for collecting cRED data on the basis of its diffraction pattern.

### 4.1. Deep convolutional neural network   

A deep convolutional neural network (CNN) (LeCun *et al.*, 1989 ▸) was used to distinguish between *good* and *bad* diffraction patterns. A basic CNN is trained to find small (in this case starting with 3 × 3 pixels) features in an image which are combined to build larger higher-level features, which may in turn be used to build even higher level features, *etc.*, depending on the number of layers in the network. The highest-level features found in the image are input into a number of dense layers, which carry out the classification, *e.g.* the features feline face, legs, torso and tail lead to the classification ‘cat’.

Image preprocessing was performed using *numpy* (Walt *et al.*, 2011 ▸) and *scikit-image* (Walt *et al.*, 2014 ▸). The primary beam is located by finding the average position of the top 5% brightest pixels in the image. A new image is cropped out from the 400 × 400 pixels around the primary beam to ensure that the strongest feature is always at the center of the image, while maintaining a consistent resolution. Because the pixels in the central beam usually have values that dwarf the values of the diffraction spots, the intensity of the pixels (*z*) was capped at the mean intensity (μ) plus four times the standard deviation (σ), *i.e.*

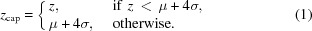
The values were then normalized by feature scaling unless the largest (*z*_max_) and smallest (*z*_min_) intensities were identical, in which case all values were set to zero, *i.e.*

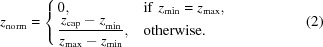
The images were finally shrunk to 150 × 150 pixels to reduce the cost of computation in the neural network.

The model was specified and trained in *Keras* (Chollet, 2015 ▸) using the *Tensorflow* (Abadi *et al.*, 2015 ▸) backend and Nvidia CUDA (Nickolls *et al.*, 2008 ▸) on approximately 78 000 labeled images of diffraction patterns split 80, 10 and 10% into training, validation and test data sets, respectively. Model details can be found in Table S1 of the supporting information. Dropout (Srivastava *et al.*, 2014 ▸) was used as regularization to avoid overfitting and rectifier activation (Hahnloser *et al.*, 2000 ▸) was used in all convolutional and dense layers, except the output layer where logistic activation was used. About 55 000 of the images were SerialED data, 15 000 cRED data and 8000 computer-generated powder rings; about 57% were labeled as *good* and 43% as *bad*. The final model was trained in batches of 75 images in 20 epochs using a dropout rate of 15%, the binary entropy as loss function, and the RMSprop optimizer on an Nvidia GeForce GTX 970 GPU. The achieved accuracies are 94.6, 93.1 and 93.3% on the training, validation and testing data sets, respectively. Note that the line between *good* and *bad* is subjective and inconsistency in the labeling probably limits the maximal accuracy.

When a diffraction pattern is passed through the CNN, a prediction score between 0.0 and 1.0 is returned, where any value greater than 0.5 corresponds to a *good* quality diffraction pattern [see also Smeets, Ångström & Olsson (2018 ▸)].

### 4.2. Application   

**Figure 2:**
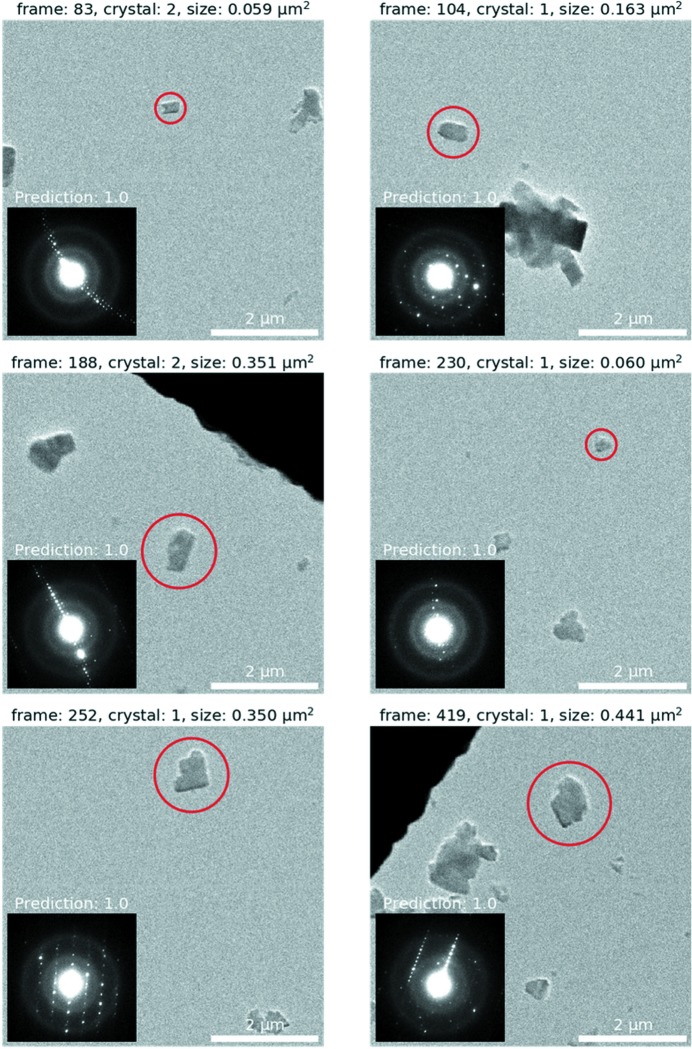
Selection of six out of 75 crystals identified by screening diffraction data acquired using the SerialED technique with a CNN. The inset in each case shows the diffraction pattern corresponding to the identified crystal circled in red. The images show an area of 5.95 × 5.95 µm.

To show the potential of the method for screening crystals, SerialED data were collected on a sample of synthetic mordenite. In total, 236 images were collected at a magnification of 2500×, by moving the stage over an area of 200 × 200 µm, and 867 diffraction patterns were collected in 24 min (corresponding to 2167 patterns per hour). The diffraction patterns are run through a script that applies the CNN algorithm and generates a csv file where each row contains the path to the image on which the crystal was identified (in imaging mode), sequence number of the image and crystal, prediction score, object size, and *x* and *y* coordinates of the sample stage. Two criteria were used to identify suitable crystals for data collection. First, only isolated crystals were selected. Crystals that were within 1.5 µm from another crystal or 0.5 µm from the edge of the image frame were discarded, leaving 75 candidates. Second, the CNN was used to predict which crystals would be most suitable, leaving 52 crystals with a prediction score of >0.5. Fig. S1 shows the almost binary distribution of the prediction scores for this data set, which means that the CNN is very confident in its prediction. The corresponding stage positions can be loaded into *Instamatic* and recalled with an accuracy of approximately ±1.0 µm, depending on the precision of the goniometer. The operator can then decide whether a crystal is indeed adequate and perform the cRED data collection experiment, or choose a different one. Six of the best crystals, as an example, are shown in Fig. 2 ▸, and six more with prediction values <1.0 in Fig. S2. This shows that the combination of complementary information from direct (images) and reciprocal (diffraction patterns) space proved to be effective for identifying suitable crystals for data collection. The time to complete the crystal screening process mainly depends on the size of the area selected, because it is limited by the speed of the stage translation on our microscope. For an area of 200 × 200 µm, the process from SerialED data collection to the final identification of suitable crystals takes about 30 min to complete.

## 5. Continuous rotation electron diffraction   

The cRED technique has been a fully manual and operator-dependent method up till now. One of the reasons was the lack of a dedicated program for data collection. Initially, cRED data were collected using the software *SoPhy* provided by ASI, normally used for setting up and calibrating the camera, using the function to collect a series of images. A typical cRED data collection experiment involves (1) unblanking the beam (if used), (2) starting the crystal rotation using the pedals while simultaneously (3) starting image recording in the camera software, and (4) tracking the crystal during rotation. On our microscope, the rotation is controlled through two pedals, one for clockwise and one for anti­clockwise rotation. The pedal needs to be held down during data collection. To complete the experiment, the following procedures are involved: stopping (1) image recording and (2) rotation, (3) saving the data on the hard drive, and (4) noting down experiment metadata, such as the starting angle, ending angle, spot number, rotation setting (rotation speed) and camera length. Manually noting metadata and saving files may lead to errors and data loss. The metadata are necessary to prepare the input files for the data processing software. Essentially all the steps, except for acquiring a series of images, were previously done manually.

Our intention is to make the cRED method more accessible and turn it into a high-throughput method. We implemented the data collection routine in the program *Instamatic* (Smeets *et al.*, 2017 ▸) and made an effort to automate as many steps as possible. In addition, we integrated a set of scripts into the program for preparing data and input files for data processing software. In this way cRED data acquisition becomes a semi-automated routine, and the number of steps required is greatly reduced. Data acquisition has been simplified through the following procedures:

(1) Start recording images automatically once rotation starts. Once the data collection routine is initialized, the program enters a state where it will wait for the stage to start rotating, which will then initiate data recording. A delay of 0.2 s is introduced to avoid acceleration and any possible backlash of the goniometer. The time and current rotation angle at the start of the experiment are logged. Data are saved to a buffer in memory and written once data collection has finished.

(2) Option to retract the beam blank automatically once rotation starts, which is especially useful for radiation-sensitive materials.

(3) Option to defocus every *n*th frame (using diffraction focus, IL1 lens) according to the needs of the operator, which is used for crystal tracking (see §5.1).

(4) Log all experimental parameters, such as exposure time, spot number, camera length, rotation speed, timestamps *etc*.

(5) Apply corrections to the diffraction data (see below), write image files with the specified formats (TIFF, SMV, MRC) and embed the required metadata where possible.

(6) Save all data to a new directory automatically. The number suffix for the directory is incremented after every experiment, so that previous data acquisitions are never overwritten.

(7) Write instruction files for data processing software, specifically *XDS* (Kabsch, 2010 ▸), *DIALS* (Winter *et al.*, 2018 ▸) and *REDp* (Wan *et al.*, 2013 ▸). The instruction files provide good default values and can be used directly, although some tweaking may be required.

The procedure for cRED data collection with *Instamatic* is illustrated in a flowchart in Fig. S3. Although the software is not yet fully automated and still requires an active operator during data collection, it can perform many of the routine steps and addresses some of the common problems with fully manual data collection. Furthermore, it introduces a standard protocol for data acquisition.

### 5.1. Crystal tracking through defocusing diffraction patterns   

The most consequential difficulty during data collection has been to keep the crystal in the beam during rotation. Crystal drift is a problem caused by goniometer mechanics and the crystal not being centered on the rotation axis, which is particularly noticeable at high tilt angles. Adjusting the height of the crystal helps to minimize the sample movements during rotation (Dierksen *et al.*, 1992 ▸; Zhang *et al.*, 2010 ▸). The problem is exacerbated because the data collection cannot be paused once the rotation has started. In our initial experiments, the position of the crystal is corrected by manual adjustment of the stage position while monitoring the shape and intensity of the diffraction pattern during data collection, and attempting to re-center the crystal once the diffraction signal becomes weak because the crystal is moving partly outside the view of the SA aperture. This is a common problem with cRED data collection, which limits the maximum rotation range that can be obtained. Gemmi *et al.* (2015 ▸) demonstrated an elegant solution with a focused electron beam, using the beam-shift deflectors to follow the pre-programmed path of the crystal, albeit with limited success.

**Figure 3:**
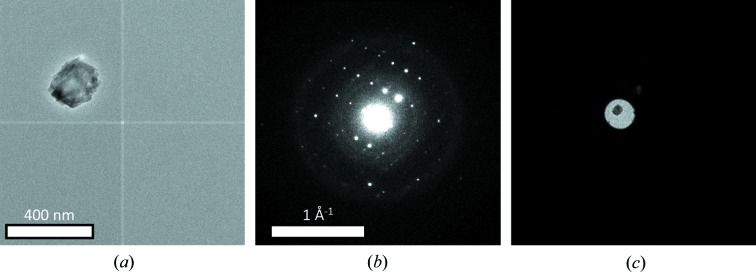
(*a*) Image taken in transmission electron microscope mode showing a crystal of mordenite (corresponding to data set 2), (*b*) diffraction pattern at 0.48° rotation and (*c*) underfocused diffraction pattern at 0.25° rotation after defocusing the IL1 lens. (*b*) and (*c*) were acquired as part of a cRED data acquisition using *Instamatic*. These images correspond to the three ray-diagram settings depicted in Fig. S4.

In our setup, improved crystal tracking is achieved by defocusing the diffraction pattern *via* the intermediate lens (IL1) at regular intervals. Crystal tracking in diffraction mode through defocusing has been described previously in the context of collecting stepwise SCED data (Wan *et al.*, 2013 ▸). In the implementation in *Instamatic*, every *n*th diffraction pattern is defocused (*n* can be tuned to the extent of the crystal movement, typically *n* = 10). This returns a snapshot of the crystal in the primary beam and can be used as a reference for tracking the crystal, simplifying the process of re-centering the crystal. At present, crystal tracking is performed manually, but the defocused images provide a way to automate tracking in the future. The ray diagrams for the different modes are illustrated in Fig. S4 and the corresponding images are given in Fig. 3 ▸. Afterwards, the diffraction focus is set back to the previous value. The defocused images are stored automatically in a different directory from the diffraction data and can be used to check and verify the data collection. Note that defocusing every *n*th image introduces gaps in the data, which may lead to partially recorded reflections and therefore reduced accuracy of the integration. Low-angle reflections are less affected by this than high-angle reflections, because they are recorded on more frames. The integration routine in *XDS*, for example, uses profile fitting to integrate the observed intensity, which means that it can compensate for missing frames to some degree. High-angle reflections that are only observed on a few frames may be lost. However, the fact that the crystal can be tracked ensures the crystal can be kept in the electron beam, which leads to higher rotation ranges, data redundancy and completeness, and, in turn, to data more suitable for structure refinements. This is demonstrated by the successful structure refinement against a data set obtained using this method in §6.

**Figure 4:**
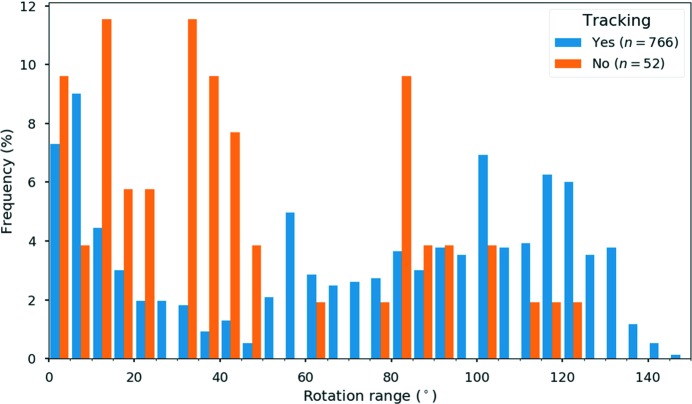
Histogram of rotation ranges achieved using *Instamatic* over 818 experiments, 766 with (in blue) and 52 without (in orange) crystal tracking.

The implementation of the crystal tracking method has had a meaningful impact on the way data are collected in our laboratory. First, it greatly reduced the number of failed or interrupted experiments that would occur because crystals moved out of the view of the SA aperture during data collection. Second, it has made it possible to consistently achieve higher rotation ranges (>120°). To follow the statistics of the cRED experiments, we implemented metadata logging for each experiment, which includes experimental information such as the rotation range, rotation speed, exposure times, camera length and number of frames collected. Fig. 4 ▸ shows a histogram of the rotation ranges achieved from 818 experiments by 15 different (including novice and experienced) operators between December 2017 and July 2018. Of these experiments, 766 (93.6%) used the crystal tracking and 50% reached high rotation ranges (>80°) so that high data completeness can be achieved, which is important for structure determination. Interestingly, the histogram reveals that *ca* 24% of all experiments were interrupted before reaching 20° rotation. This is usually the result of the crystal moving out of the beam. The spike around 60° can be explained by the use of a cooling or cryo-transfer specimen holder, which has a limited rotation range (±42°). The data show that the crystal tracking implementation has contributed to the speed and success rate of the cRED data collection. In turn, this has increased the reproducibility of the experiments and made the method more accessible to novice and experienced users alike.

### 5.2. Other practical aspects for cRED data collection   

Several other important aspects need to be considered during cRED data collection: (1) timing, (2) lens hysteresis and (3) possible primary beam shift after defocusing. First, because the goniometer keeps rotating throughout the entire data collection, including the gap periods for crystal tracking under which no diffraction patterns are recorded, the timing of a defocus cycle must match the acquisition time of a diffraction pattern. Gaps in the data must cover the same rotation range (or an integer multiple thereof) to ensure that the oscillation angle of the missing data is consistent with the rest for the data processing algorithms implemented in *XDS* (Kabsch, 2010 ▸) and *DIALS* (Winter *et al.*, 2018 ▸). Another point related to timing is that any changes to the electron beam optics are not instantaneous. We estimate the time it takes for the electron beam to come back to its refocused state to be around 300–400 ms, although this number goes up when a larger defocus value is applied. The data collection routine keeps track of the average acquisition time for a diffraction pattern, which is a summation of the exposure time (typically 500 ms), readout time (8 ms for the Timepix camera) and overhead, for example for allocating memory and arranging the data (approximately 3–4 ms). Each defocused image is taken with a much shorter exposure time (typically 10 ms), so that approximately 400 ms can be allocated to refocus the diffraction pattern, taking into account that every call to the JEOL API takes about 35 ms. Second, it is important to relax the beam before the experiment, because frequently changing the value of the intermediate lens introduces a hysteresis that influences the position of the primary beam in the diffraction pattern on our transmission electron microscope. This may cause the position of the primary beam to drift after a defocus cycle. To avoid this, the electron beam is relaxed by toggling between the focused and defocused state a few times before the data collection. In this way, the primary beam is set to its neutral position. Lastly, depending on the alignment of the microscope and the position of the SA aperture, the defocus is not necessarily applied concentrically. In combination with the fact that refocusing is not instantaneous, this can manifest itself as a small but noticeable shift of the primary beam position in the first pattern following a defocus cycle. The shifts are typically less than 0.2 pixels, which did not cause any issues with data processing (§S1).

### 5.3. Data processing   

The steps for data processing have been adapted from the method described by Smeets, Zou & Wan (2018 ▸). Because the pixels connecting the four modules (each 256 × 256 pixels in size) in the Timepix detector are three times larger (165 µm instead of 55 µm), the images are converted to a 516 × 516 array to ensure the correct geometry for further processing [see also Nederlof *et al.* (2013 ▸)]. The cross pixels are masked in *XDS* using the UNTRUSTED_RECTANGLE instruction. A flatfield correction is applied by *Instamatic* to correct for slight variations in pixel response, which also partially accounts for the effects of the larger pixels between the modules. The position of the primary beam is estimated at the pixel with the largest intensity value on the diffraction pattern after applying a Gaussian filter with a large enough standard deviation (usually 10–30). The median value for the primary beam positions over all diffraction patterns is used in the data processing software packages (*XDS* and *DIALS*), which expect a stationary primary beam. In *XDS*, an affine transformation can be applied to each image using the X-GEO_CORR/Y-GEO_CORR instructions to correct for the lens distortions (Capitani *et al.*, 2006 ▸). An elliptical distortion with an eccentricity of 0.22 was observed on our microscope, and the required geometrical correction files for *XDS* are generated by *Instamatic*. Missing diffraction patterns (as a result of tracking) are specified in the *XDS* input file using the EXCLUDE_DATA_RANGE instruction and in *DIALS* using the scan_range (for dials.find_spots) and exclude_images (for dials.integrate) command-line parameters. The oscillation angle used for integration is calculated by dividing the rotation range by the data collection time.

## 6. Application for structure analysis of mordenite   

The crystal tracking method has been applied for cRED data collection and structural analysis using a well known synthetic zeolite with the mordenite structure (Meier, 1961 ▸) as an example (Fig. S5). Firstly, we collected two cRED data sets using *Instamatic* with the conditions presented in Table 1 ▸, defocusing every tenth image for crystal tracking. The data were processed using the software *XDS* (Kabsch, 2010 ▸) and indexed using the lattice parameters of *a* = 18.668, *b* = 20.513, *c* = 7.691 Å, α = 89.93, β = 90.31, γ = 89.59° for data set 1, and *a* = 18.619, *b* = 20.838, *c* = 7.753 Å, α = 90.20, β = 90.12, γ = 90.52° for data set 2. Both fit with the expected orthorhombic *C*-centered unit cell of mordenite and are close to the published lattice parameters (*a* = 18.13, *b* = 20.49, *c* = 7.52 Å). Reflection intensities were extracted in space group *Cmcm* (Table 2 ▸) using *XDS*. We noticed that data set 2 is of higher quality than data set 1, with a higher mean 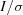 of unique reflections (6.25 *versus* 2.37) and lower redundancy-independent *R* factor, *R*_meas_ (10.8% *versus* 33.0%), despite having a lower completeness (93.6 *versus* 99.3%). The difference may be attributed to the choice of crystal or to the choice of SA aperture. For data set 2, a smaller aperture was used than for data set 1. A smaller SA aperture does not reduce the dose on the crystal, but can prevent unwanted local information and (inelastic) scattering events that contribute to increased background and noise levels (Fig. S6).

For both data sets, the structure could be determined by using direct methods implemented in *SHELXS* (Sheldrick, 2008 ▸). All framework Si and O atoms were found successfully in the initial model from the structure solution. *SHELXL* (Sheldrick, 2008 ▸, 2015 ▸) was used for structure refinement, using the known unit-cell parameters from the literature (Meier, 1961 ▸). All Si and O atoms were refined anisotropically. While there is no need to use any restraints for data set 2, for data set 1, rigid-bond restraints (Thorn *et al.*, 2012 ▸) were applied to all framework atoms using the RIGU instruction to maintain reasonable atomic displacement parameters. In addition, the resolution was cut to 0.91 Å for data set 1, because of the low 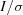 for reflections with *d* < 0.91 Å. Finally, we introduced an extinction coefficient (EXTI), which is an empirical correction useful when some of the most intense reflections have reduced the intensities, for example as a result of dynamical scattering (see also §S3). We were unable to find any sodium cations or water molecules in the difference potential map. The details of the refinement are given in Table 3 ▸. The difference in data quality is reflected in the precision of the structure refinement, where the *R*1 value for data set 1 (*R*1 = 30.07%) is significantly higher than that for data set 2 (*R*1 = 17.69%). The geometry of the distances and angles for both refined structures was analyzed by using *PLATON* (Spek, 2009 ▸; Tables 4 ▸ and S2–S4).

**Figure 5:**
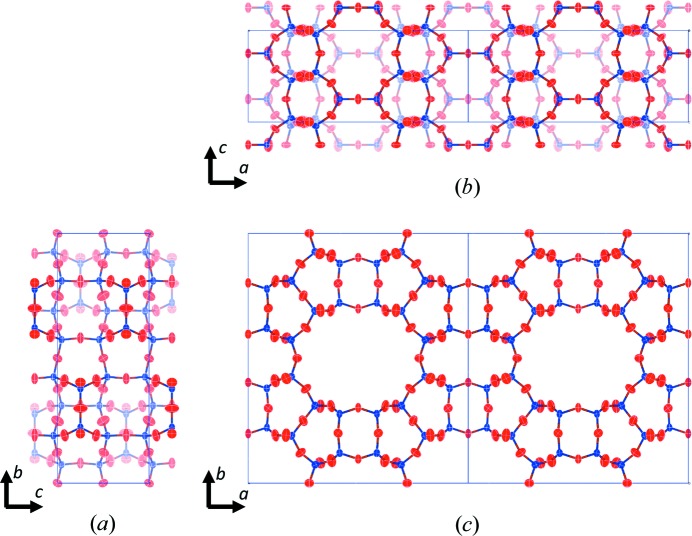
(*a*)–(*c*) Refined structure of mordenite from data set 2, showing atomic displacement parameters for the Si and O atoms at the 50% probability level along the *a*, *b* and *c* axis, respectively.

In the absence of restraints on the structure parameters, the spread of the Si—O bond distances is 1.59–1.66 Å (mean: 1.610 ± 0.018 Å) for data set 1 and 1.59–1.64 Å (mean: 1.614 ± 0.012 Å) for data set 2. The tetrahedral O—Si—O angles are 107.2–113.0° (mean: 109.5 ± 1.8°) for data set 1 and 106.4–112.4° (mean: 109.5 ± 1.9°) for data set 2. The values for both data sets are consistent with the expected values of *d*(Si—O) = 1.61 ± 0.01 Å and ∠(O—Si—O) = 109.5 ± 0.8°. Compared with the published structure of mordenite we obtained accurate refined results for both data sets (Tables S2–S4). Particularly noteworthy are the atomic displacement parameters obtained for data set 2 (Fig. 5 ▸). Anisotropic displacement parameters are known to act as a fudge factor for poor quality data, resulting in physically meaningless displacement ellipsoids. For data set 2, however, the atomic displacement parameters are physically sensible. The atomic displacement parameters for oxygen are slightly larger than those for Si, and elongated perpendicular to the plane formed by the Si—O—Si bond. All bonds pass the Hirshfeld rigid-bond test (Hirshfeld, 1976 ▸), with an r.m.s. difference of 0.0058 Å. The largest differences are found for the Si3—O1 and Si3—O4 bonds, with values of 0.010 (5) and 0.013 (8) Å, respectively (Table S6). This is approximately an order of magnitude larger than the value of 0.001 Å suggested by Hirshfeld for X-ray diffraction data. This may indicate that the precision of the structure refinement using cRED data is not yet at a level where such small deviations may be discerned. We therefore consider the atomic displacement parameters to be reliably determined, but further study is warranted.

**Figure 6:**
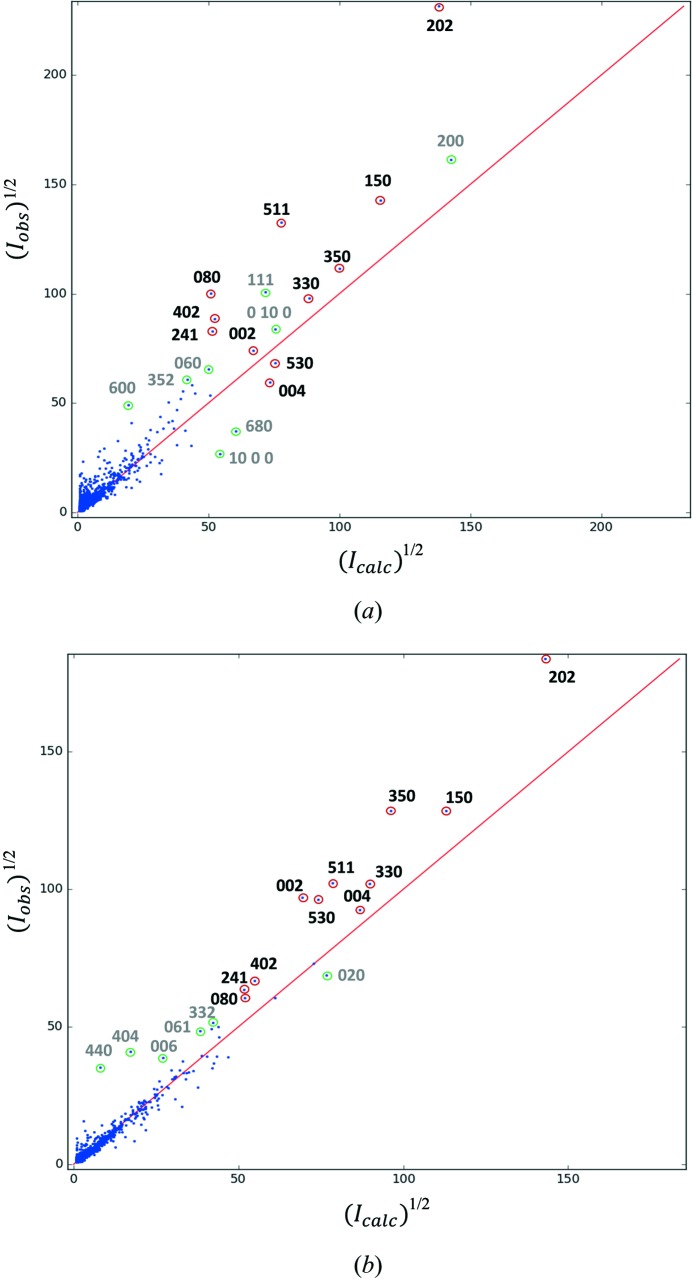
*F*_obs_ *versus F*_calc_ plots for mordenite from (*a*) data set 1 and (*b*) data set 2. Common notable outlier reflections are circled in red and other outliers in green. Plots were generated using the program *ANAFCF* (Lutz & Schreurs, 2012 ▸).

After the model was parametrized the data were examined for outliers. An *F*_obs_–*F*_calc_ plot (Fig. 6 ▸) was created to obtain a visual impression of the data quality using the software *ANAFCF* and *LOGLOG* (Lutz & Schreurs, 2012 ▸). An fcf file of phased structure factors containing *h*, *k*, *l*, *F*_obs_, σ(*F*_obs_), A(real) and B(imag) from *SHELXL* (LIST 4) was used to prepare the plots. For data set 1, the majority of the most discrepant reflections belong to the (*hk*0) plane, which can be attributed to the rotation almost exactly passing through the [00*l*] zone axis, where the dynamical effect is maximized. Notwithstanding, the two plots are mutually consistent (Fig. S8), and they contain mostly the same reflections which are roughly proportionate to one another.

### 6.1. Validating crystal tracking   

**Figure 7:**
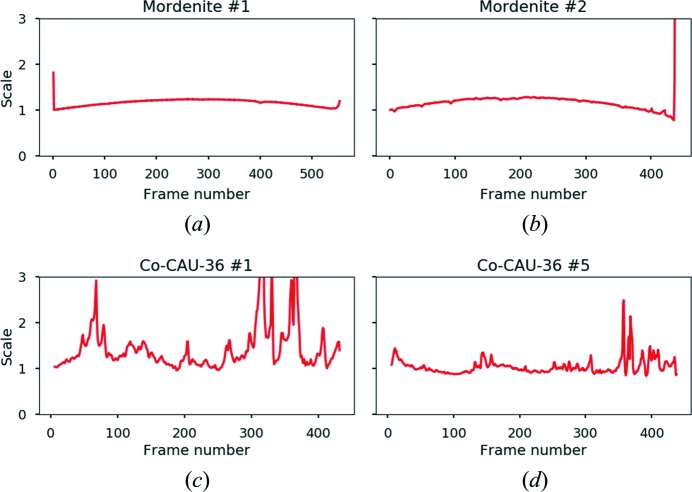
Normalized scaling factors from diffraction patterns collected for (*a*), (*b*) mordenite (this study) and (*c*), (*d*) Co-CAU-36 (Wang *et al.*, 2018 ▸) as calculated by *XDS* (SCALE in file INIT.Lp) can be used to judge the tracking of the crystals. If the crystal moves (partially) out of the SA aperture, the image scale is affected.

To judge how well the crystal remains in the SA aperture during data collection, the SCALE factor reported by the *INIT* job in *XDS* (Kabsch, 2010 ▸) can be consulted. The SCALE factor uses the ED frames only and is employed in *XDS* to correct for variations in the incident beam flux. We found that this parameter is sensitive to the crystal moving (partially) out of the SA aperture. As the crystal gets obscured by the SA aperture, the corresponding diffracted intensities weaken. Figs. 7 ▸(*a*) and 7 ▸(*b*) show the evolution of the scale factor with frame number for data sets 1 and 2, respectively. The data reveal a slowly varying scale over the entire image range, indicating that the crystal remained in the aperture during data acquisition. The large spike on the right of Fig. 7 ▸(*b*) corresponds to the diffraction data being obscured by the copper grid at a high rotation angle. It is unclear what happened for the first frame in Fig. 7 ▸(*a*).

For comparison, the scale evolution for two data sets (out of eight) from a previous study on the coordination polymer Co-CAU-36 (Wang *et al.*, 2018 ▸) is given in Figs. 7 ▸(*c*) and 7 ▸(*d*). cRED data on Co-CAU-36 were collected while blindly tracking the position on the crystal (*i.e.* before the defocus method was implemented). Although the data sets consist of rotation ranges of over 100°, in both cases the crystal repeatedly moved (partially) outside the view of the SA aperture, as indicated by the rapidly varying SCALE factor. In the Co-CAU-36 study, data set 5 (Fig. 7 ▸ *d*) was found to comprise the highest data quality, and was used to determine and refine the crystal structure. Data set 1 (Fig. 7 ▸ *c*) was still sufficient for determination of the crystal structure.

## 7. Conclusions   

We have shown that high-throughput SCED data collection of submicrometre-sized crystals using the continuous rotation method is attainable through software automation, as implemented in the program *Instamatic*. This is achieved on two fronts. First, a routine for the screening of suitable crystals was developed, making use of the SerialED method to collect image and diffraction data on a large number of crystals. The image data are used to find isolated crystals. A CNN was trained to differentiate between *good* and *bad* diffraction patterns and identify the most promising crystals. Combining direct (image) and reciprocal (diffraction) space information in this way was found effective for identifying suitable crystals on which to collect cRED data.

Second, we have automated many of the steps to collect cRED data in *Instamatic*, and some of the problems with data collection have been addressed. Of particular importance is that the crystal can be tracked during the data collection by defocusing the diffraction pattern at regular intervals, which enables reliable and reproducible experiments. This also makes high rotation ranges more accessible, so that the data cover a larger portion of reciprocal space. Moreover, the collected data format is compatible with standard single-crystal processing software like *XDS*, *DIALS* and *REDp*, and usable input files with compatible data files are produced by *Instamatic*. All these factors make the method more accessible to novice and irregular users, and enable data to be collected routinely in under 5 min.

The data show that, despite forgoing every *n*th frame for the purpose of crystal tracking, the resulting data set can be of high quality and suitable for structure refinement. The accuracy of the refined structure was assessed by examining the deviations in the bond lengths and angles. The atomic displacement parameters for data set 2 were refined anisotropically and validated by means of the Hirshfeld rigid-body test, showing that physically meaningful atomic displacement parameters can be obtained from cRED data. This opens up new possibilities to study atomic motion (libration, translation, internal vibrations) and disorder (static or dynamic) from submicrometre-sized crystals.

At this stage, cRED data collection still requires an active operator to supervise the data collection and correct for the position of the crystal during the experiment. The development of automated tracking procedures using the defocused images is currently in progress. In the future, we hope to further integrate the SerialED and cRED methods for automated crystal selection and data collection so that a large number of data sets can be collected without (or with very little) human supervision. With the increased interest in radiation-sensitive materials, such as organic, pharmaceutical and macromolecular crystals, more automation is a way to reduce the dose on a sample. The methods described here are generally applicable and can be applied to any material that forms submicrometre-sized crystals.

The software used to collect the data is available from http://github.com/stefsmeets/instamatic. Movies of the data collection using crystal tracking and the crystallographic data for both structures in CIF format are provided as supporting information. The cRED and SerialED data sets used in this study have been deposited at http://dx.doi.org/10.5281/zenodo.1321880.

## Supplementary Material

Crystal structure: contains datablock(s) mordenite_1, mordenite_2. DOI: 10.1107/S1600576718015145/yr5038sup1.cif

Structure factors: contains datablock(s) mordenite_1. DOI: 10.1107/S1600576718015145/yr5038mordenite_1sup2.hkl

Structure factors: contains datablock(s) mordenite_2. DOI: 10.1107/S1600576718015145/yr5038mordenite_2sup3.hkl

Movie of the data collection using crystal tracking. DOI: 10.1107/S1600576718015145/yr5038sup4.mp4

Movie of the data collection using crystal tracking. DOI: 10.1107/S1600576718015145/yr5038sup5.mp4

Supporting information file. DOI: 10.1107/S1600576718015145/yr5038sup6.pdf

The cRED and SerialED data sets used in this study URL: http://dx.doi.org/10.5281/zenodo.1321880

CCDC references: 1875576, 1875577

## Figures and Tables

**Table 1:** Experimental details for the cRED data collection of the two data sets of mordenite

	Data set 1	Data set 2
λ (Å)	0.02508 (200 keV)	0.02508 (200 keV)
Oscillation angle (°)	0.2314	0.2336
Tilt range (°)	−64.06 to 63.91 (127.97)	−43.90 to 58.65 (102.55)
Frames used†	554	430
No. of images in between frames	55	43
Defocus for an image interval‡ (exposure time)	19 993 (*t* = 0.01 s)	20 693 (*t* = 0.0 1 s)
Exposure time per frame (s)	0.5	0.5
Acquisition time per frame (s)	0.512	0.512
Total acquisition time (s)	283.0	224.7
Spot size	2	2
Effective aperture radius (µm)	0.75	0.35
Camera length (mm)	250	250

† The last few frames from data set 2 were excluded, because they were obscured by the copper grid.

‡ A defocused image was taken every tenth diffraction pattern.

**Table 2:** Data processing details using *XDS* for the two data sets of mordenite

	Data set 1	Data set 2
Resolution (Å)	0.80	0.80
No. of total reflections	6804	5244
No. of unique reflections	1665	1585
Completeness (%)	99.3	93.6
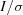	2.37	6.25
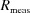 (%)	33.0	10.8
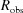 (%)	28.5	8.8
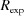 (%)	28.1	8.7

**Table 3:** Crystallographic details for the refinement of the two data sets of mordenite

	Data set 1	Data set 2
Chemical formula (refined)	Si_48_O_96_	Si_48_O_96_
Space group	*Cmcm* (63)	*Cmcm* (63)
*a* (Å)	18.110	18.110
*b* (Å)	20.530	20.530
*c* (Å)	7.528	7.528
Resolution (Å)	0.91	0.80
No. of total reflections	4432	5244
No. of unique reflections (all)	1090	1585
No. of unique reflections [*F*_o_ > 4σ(*F*_o_)]	684	1140
Refined parameters	96	96
Restraints	93	0
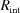	0.2658	0.0878
*R*1 for *F*_o_ > 4σ(*F*_o_)	0.2841	0.1602
*R*1 for all data	0.3007	0.1769
Goodness of fit	1.626	1.610

**Table 4:** Refined framework bond distances and angles of mordenite Values in parentheses are errors on the least significant digits.

		Data set 1			Data set 2		
	Nominal value	Min.	Max.	Average	Min.	Max.	Average
*T*—O (Å)	1.61	1.59	1.66	1.610 (18)	1.59	1.64	1.614 (12)
O—*T*—O (°)	109.5	107.2	113.0	109.5 (18)	106.4	112.4	109.5 (19)
*T*—O—*T* (°)	145.0	142.4	180.0	154.0 (115)	143.5	180.0	153.3 (120)
