# Supplementary material for: High-throughput continuous rotation electron diffraction data acquisition via software automation
Source: J Appl Crystallogr. 2018 Nov 22;51(Pt 6):1652–61. doi: 10.1107/S1600576718015145 (PMC6276279; doi:10.1107/S1600576718015145)
Supplement: Supplementary file 6 [file j-51-01652-sup6.pdf]

JOURNAL OF APPLIED CRYSTALLOGRAPHY

Volume 51 (2018)

Supporting information for article:

High-Throughput Continuous Rotation Electron Diffraction Data Acquisition Via Software Automation

Magdalena Ola Cichocka, Jonas Ångström, Bin Wang, Xiaodong Zou and Stef Smeets

S1. Primary beam position

To evaluate the effect of defocusing every n^{th} frame on the data, the positions of the primary beam for both data sets were calculated using the S*hift Evaluation* routine implemented in the program *REDp* (Wan *et al.*, 2013), and shown in Figure S7. This revealed a small drift of the primary beam for dataset 1 in Figure S7A (*X*-coordinate), indicating that the primary beam position was not fully neutralized before the start of the experiment. The *X*- and *Y*-coordinates for dataset 2 (Figure S7B) show a sharp change in the position of the primary beam in the last few frames, which can be attributed to the sample being obscured by the copper grid at a high rotation angle. Accordingly, these frames were excluded from the integration step in *XDS*. Second, depending on the alignment of the microscope and the position of the SA aperture, the defocus is not necessarily applied concentrically. Together with the fact that the defocus is not applied instantaneously, this typically manifests itself as a small shift of the primary beam position in the first frame after the defocus has been applied. This can be seen in Figure S7B for the *X*-coordinate, where the primary beam shifts about 0.1-0.2 pixel from the first to the second frame after defocus. Overall, the position of the primary beam is remarkably stable and varies only within a few tenths of pixels over both datasets.

S2. Geometry for the refined structure of mordenite

Typically, zeolites have the Si-O distance between 1.56-1.66Å, the Si-O-Si angles >135° and the O-Si-O angles between 105-114°. Tables S2-S4 show the geometry of the refined structures. The published data (Meier, 1961) are shown for comparison. Tables S5 and S6 show the Hirshfeld rigid-bond test values.

S3. Effect of the extinction coefficient (EXTI)

We have also checked the F_{obs}–F_{calc} plot when before introducing an extinction coefficient (EXTI) to the refinement, to better understand its effect on the refinement. Figure S9 shows a few low-index outliers that lie mainly on the right side of the red line, where $F_{calc}>F_{obs}$. This could mean that there is remaining residual electrostatic potential that has not been accounted for in the model. For porous materials, this is usually attributed to extra-framework species included in the channels and cavities. Mordenite is synthesized using sodium ions, and it is well known that zeolites absorb water from the atmosphere. The presence of extra-framework species strongly affects the low-index reflections, but we were unable to locate any water or sodium ions in the channels. This may mean that the water molecules are not ordered in the crystal structure, or that they are removed during the measurement by being exposed to high vacuum and electron radiation. Attempts to model this as diffuse solvent using the SWAT parameter were not successful. Another reason can be dynamical scattering events, which tend to dampen strong reflections. The extinction coefficient in part corrects for these factors, lowering the agreement values, and in the process shifts the marked outliers to the left side of the red line as shown

in Figure S9. We believe that the EXTI parameter is therefore best to be introduced only at the very end of the refinement.

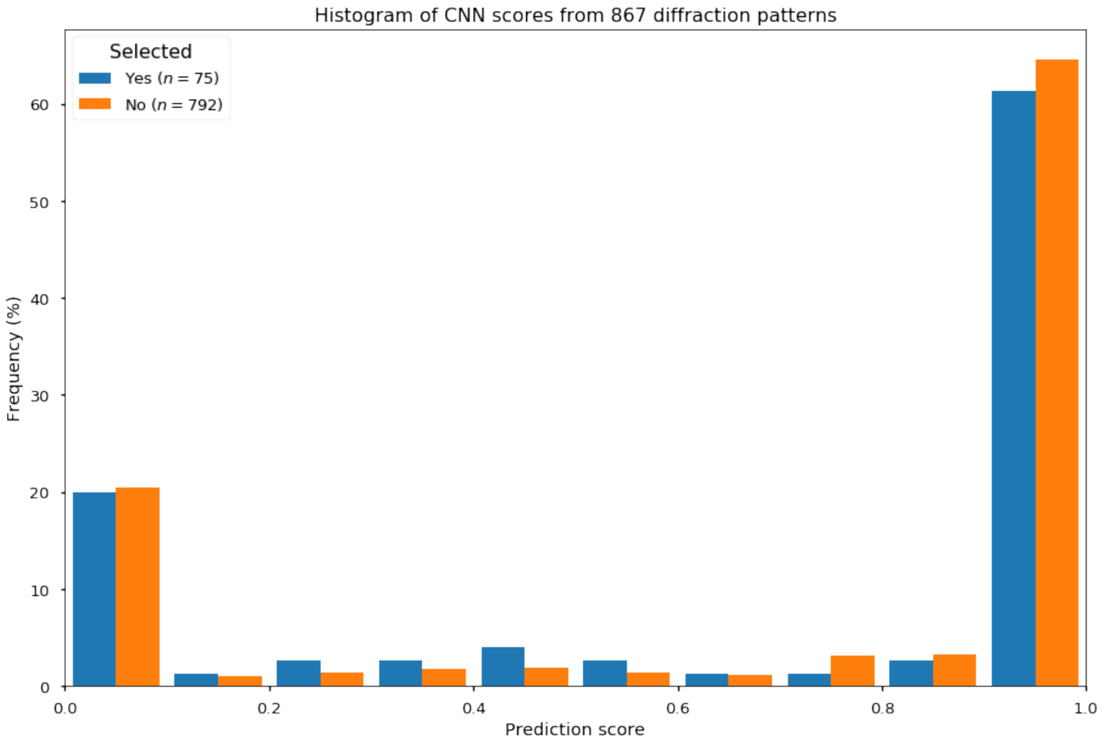

Figure S1 Histogram with the distribution of scores from the CNN over 867 diffraction patterns. The blue bars correspond to the crystals that were selected based on the distance criteria, whereas the orange bars correspond to all other diffraction patterns.

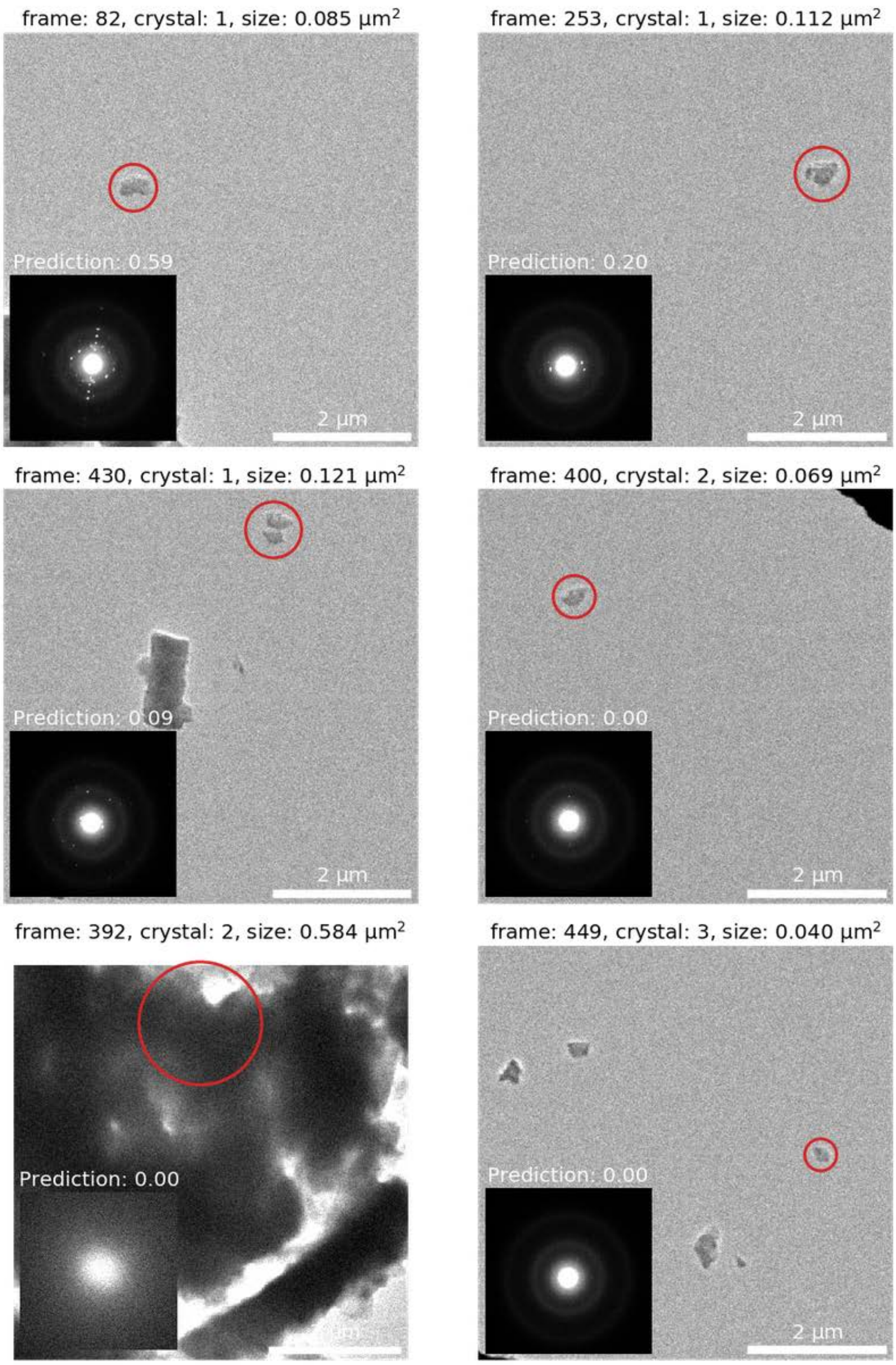

Figure S2 Selection of six out of 75 crystals identified by screening diffraction data acquired using the SerialED technique with a CNN. The inset shows the diffraction pattern corresponding to the identified crystal circled in red. The images show an area of 5.95 × 5.95 µm.

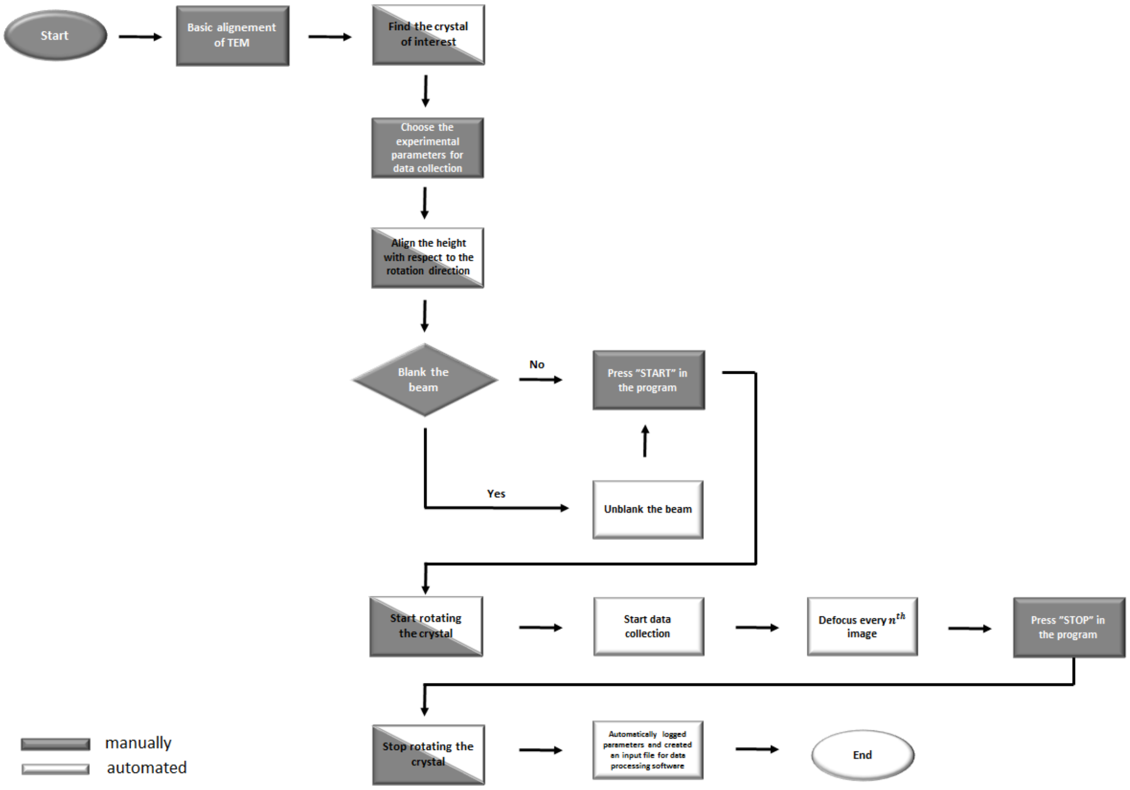

Figure S3 Flowchart illustrating the basic cRED data collection procedure. The grey and white colour indicates manual and automated part, respectively.

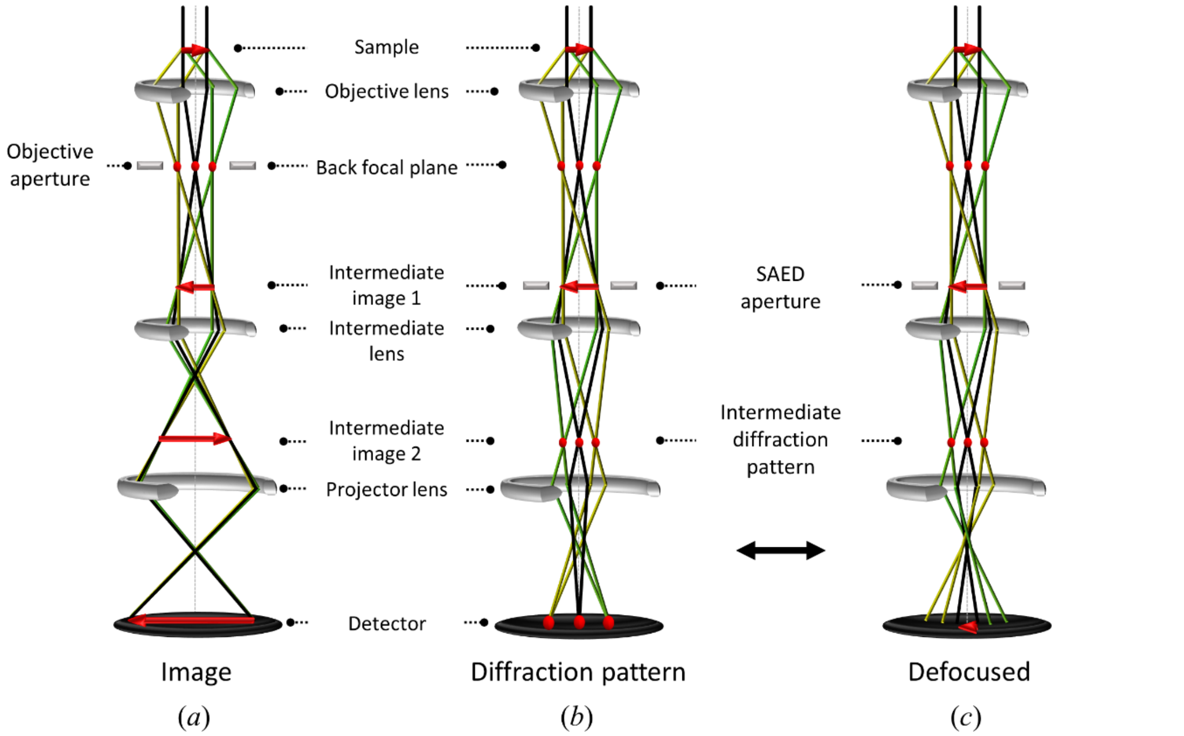

Figure S4 Schematic representation of the ray paths in (*a*) image mode, (*b*) diffraction mode and (*c*) defocused diffraction pattern for crystal tracking.

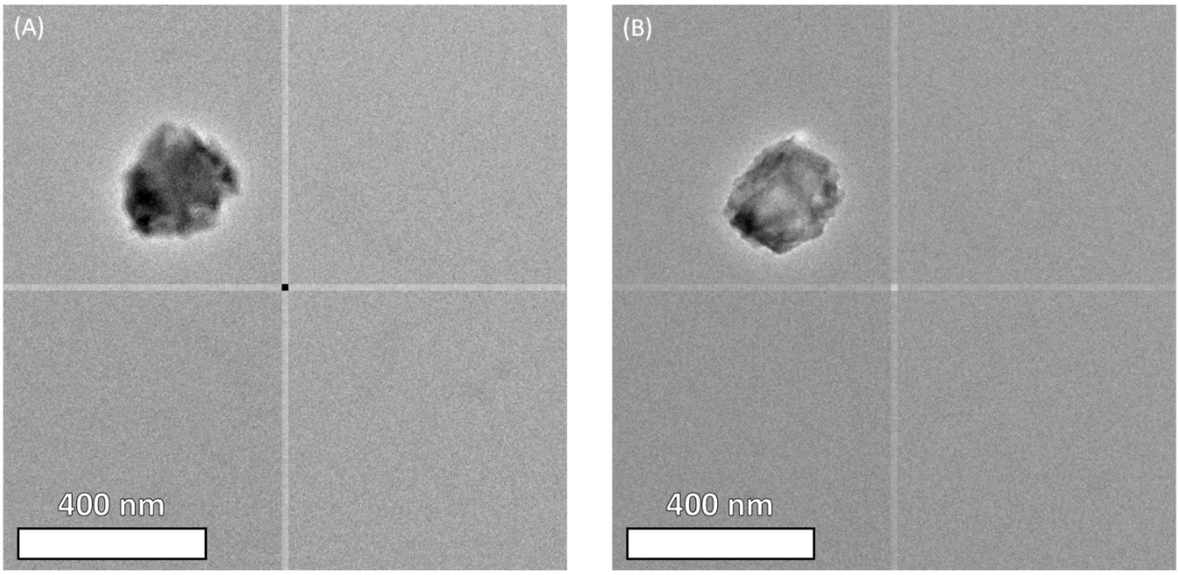

Figure S5 TEM images showing the morphology of the mordenite crystals corresponding to (A) dataset 1 and (B) dataset 2.

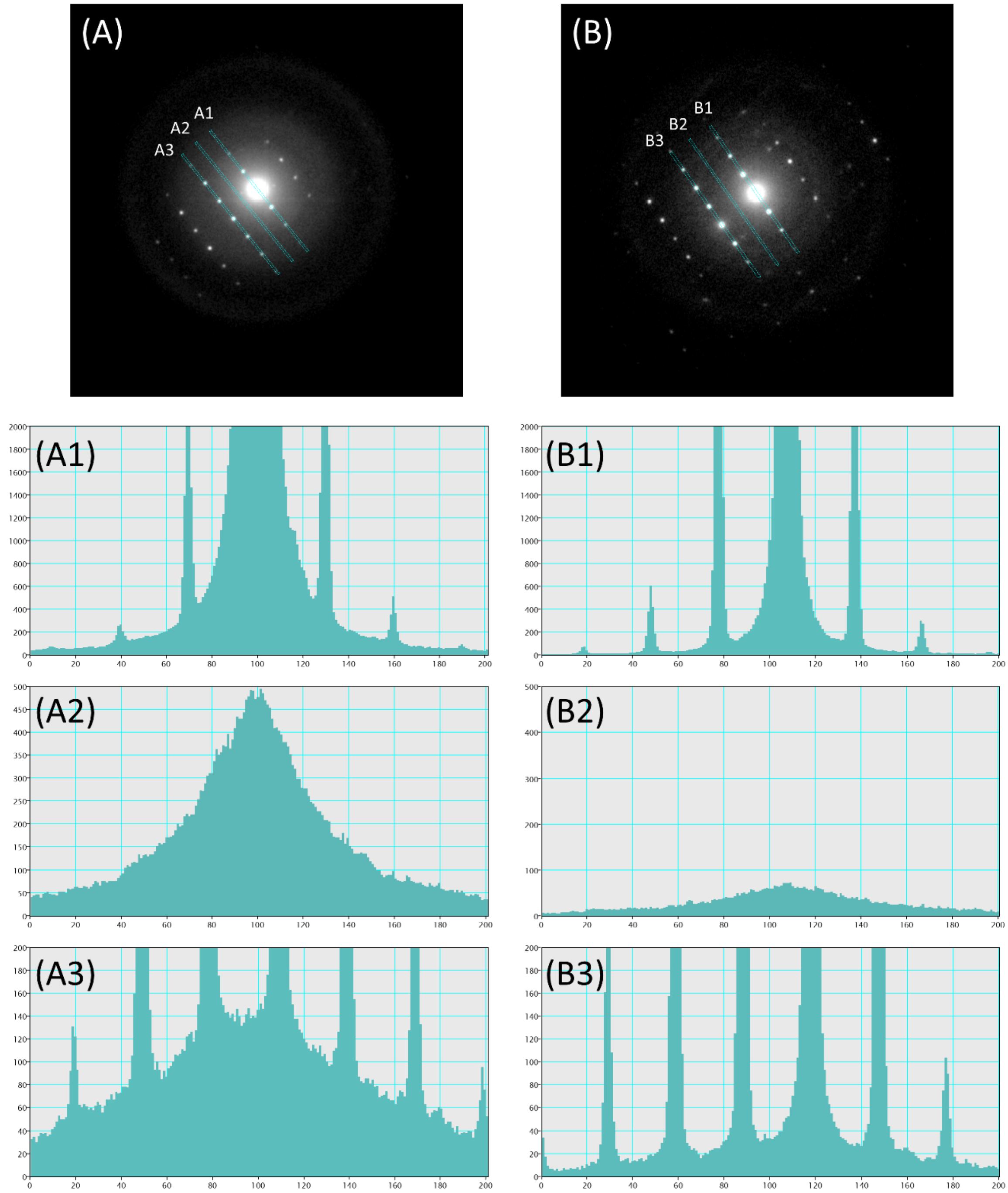

Figure S6 Selected electron diffraction patterns from (A) dataset 1 (frame 122) and (B) dataset 2 (frame 168) with the corresponding integrated intensity profiles (A1-A3, B1-B3) from the highlighted areas comparing the background levels.

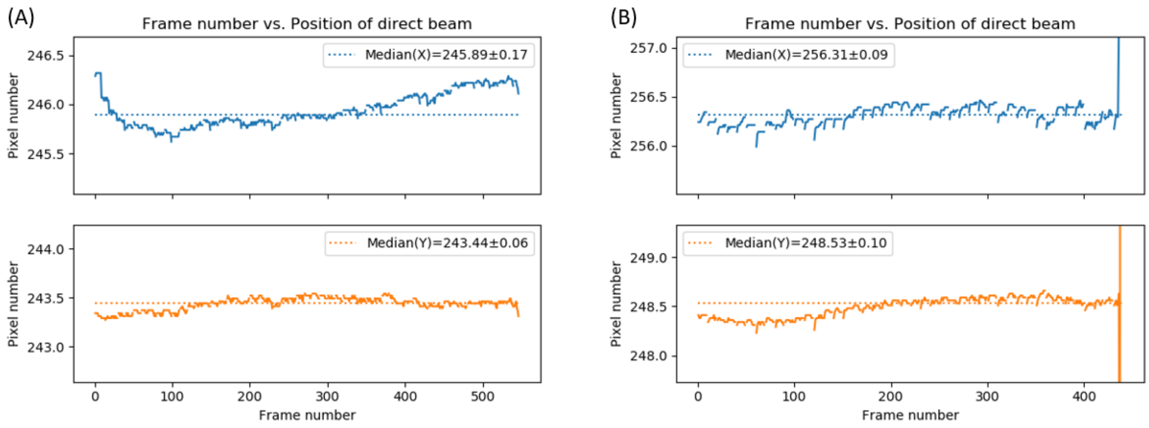

Figure S7 Variation of the position of the primary beam (*x* and *y* pixel position) with respect to the frame number for (A) dataset 1, and (B) dataset 2.The primary positions were calculated using the *Shift Evaluation* function implemented in the *REDp* software (Wan *et al.*, 2013).

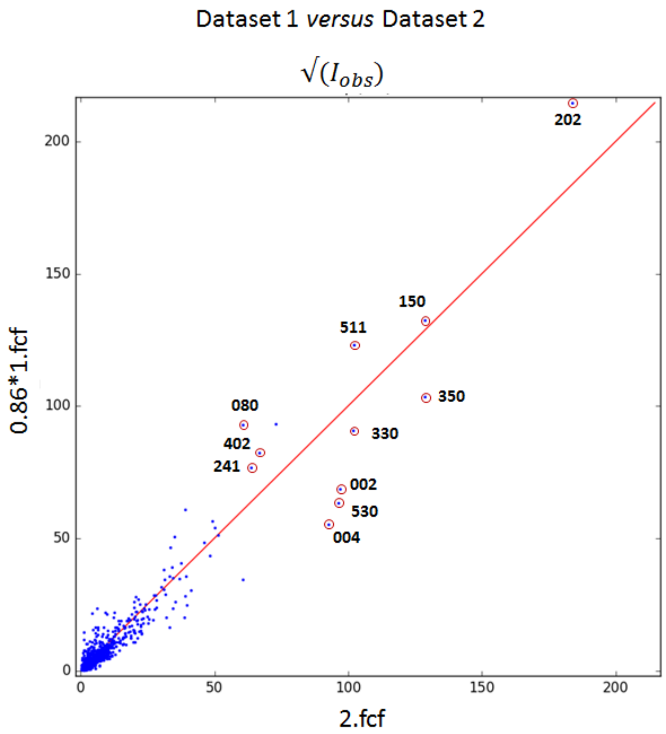

Figure S8 A comparison of F_{obs} vs. F_{calc} plot for a zeolite mordenite from dataset 1 and 2. Common notable outlier reflections are circled in red. The plot was generated using the program *loglog* (Lutz &

Schreurs, 2012).

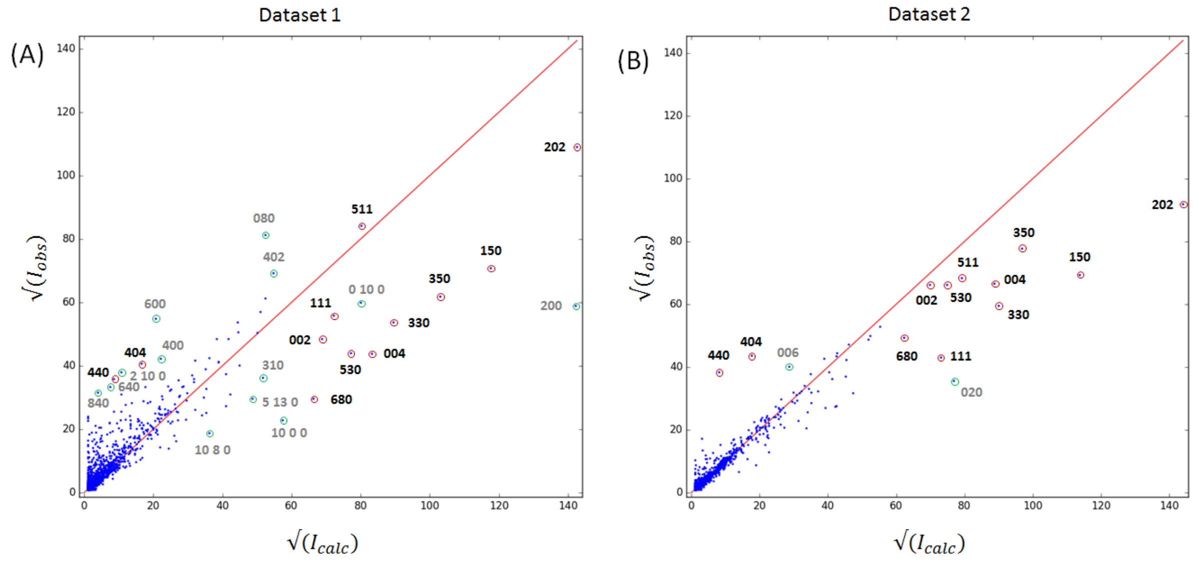

Figure S9 F_{obs} vs. F_{calc} plots for a zeolite mordenite from (A) dataset 1 and (B) dataset 2 showing the correlation before an extinction coefficient (EXTI) was introduced to the refinement. Common notable outlier reflections are circled in red, and other outliers in green. Plots were generated using the program *anafcf* (Lutz & Schreurs, 2012).

Table S1 Design of the neural network.

Layer (type)	Output shape	activation	weights shape + offset
2D convolutional (3x3)	(148, 148, 64)	rectifier	(3, 3, 1, 64) + 64
max pooling (2x2)	(74, 74, 64)	-	-
2D convolutional (3x3)	(72, 72, 64)	rectifier	(3, 3, 64, 64) + 64
max pooling (2x2)	(36, 36, 64)	-	-
2D convolutional (3x3)	(34, 34, 64)	rectifier	(3, 3, 64, 64) + 64
max pooling (2x2)	(17, 17, 64)	-	-
2D convolutional (3x3)	(15, 15, 64)	rectifier	(3, 3, 64, 64) + 64
max pooling (2x2)	(7, 7, 64)	-	-
2D convolutional (3x3)	(5, 5, 64)	rectifier	(3, 3, 64, 64) + 64
Flatten and dropout	1600	-	-
Dense (64 nodes)	64	rectifier	(1600, 64) + 64
Dense (64 nodes)	64	rectifier	(64, 64) + 64
Dense (1 node)	1	logistic	(64, 1) + 1

Table S2 Refined Si-O bond distances for mordenite structure from dataset 1, 2 and the reference structure (Meier, 1961).

	**Dataset 1**	**Dataset 2**	**Meier (1961)**
Si(1) - O(1)	1.606(16)	1.610(6)	1.628
Si(1) - O(3)	1.592(15)	1.617(6)	1.614
Si(1) - O(6)	1.614(9)	1.612(4)	1.645
Si(1) - O(7)	1.655(9)	1.638(4)	1.629
Si(2) - O(2)	1.589(16)	1.612(6)	1.604
Si(2) - O(3)f	1.617(15)	1.599(6)	1.619
Si(2) - O(5)	1.613(10)	1.615(4)	1.618
Si(2) - O(8)	1.603(8)	1.604(3)	1.607
Si(3) - O(1)	1.590(16)	1.621(6)	1.623
Si(3) - O(1)c	1.591(16)	1.621(6)	1.589
Si(3) - O(4)	1.628(19)	1.642(10)	1.647
Si(3) - O(9)	1.598(11)	1.605(5)	1.632
Si(4) - O(2)	1.603(17)	1.613(6)	1.633
Si(4) - O(2)c	1.603(17)	1.613(6)	1.605
Si(4) - O(4)	1.632(19)	1.592(10)	1.645
Si(4) - O(10)	1.631(11)	1.614(5)	1.629
**Average**	**1.610(18)**	**1.614(12)**	**1.623(19)**
**Min**	**1.590**	**1.592**	**1.589**
**Max**	**1.655**	**1.642**	**1.647**

Symmetry operations referred to the coordinates: c= x,y,1/2-z; f= 1/2-x,1/2-y,1-z

Table S3 Refined O-Si-O angles for mordenite structure from dataset 1, 2 and the reference structure (Meier, 1961).

	**Dataset 1**	**Dataset 2**	**Meier (1961)**
O(1) - Si(1) - O(3)	112.3(9)	111.9(4)	113.3
O(3) - Si(1) - O(6)	111.2(9)	110.5(5)	111.5
O(2) - Si(2) - O(5)	107.7(9)	108.1(4)	105.9
O(5) - Si(2) - O(8)	111.1(8)	109.8(4)	110.7
O(1) - Si(3) - O(4)	108.5(8)	110.7(4)	111.3
O(4) - Si(3) - O(9)	112.8(11)	112.4(6)	113.4
O(2) - Si(4) - O(4)	108.1(7)	111.2(4)	111.7
O(4) - Si(4) - O(10)	113.0(11)	112.3(6)	113.0
O(1) - Si(1) - O(6)	109.1(9)	108.1(4)	106.5
O(3) - Si(1) - O(7)	107.2(9)	106.4(4)	105.4
O(2) - Si(2) - O(8)	109.7(6)	111.2(3)	110.5
O(5) - Si(2) - O(3)f	108.3(9)	109.2(5)	109.3
O(1) - Si(3) - O(9)	108.0(8)	106.3(4)	105.2
O(4) - Si(3) - O(1)c	108.5(8)	110.7(4)	111.3
O(2) - Si(4) - O(10)	107.4(7)	106.9(4)	105.6
O(4) - Si(4) - O(2)c	108.1(7)	111.2(4)	111.7
O(1) - Si(1) - O(7)	108.7(8)	109.6(4)	109.6
O(6) - Si(1) - O(7)	108.2(7)	110.4(4)	110.6
O(2) - Si(2) - O(3)f	109.6(9)	107.5(4)	109.2
O(8) - Si(2) - O(3)f	110.4(8)	110.9(3)	111.0
O(1) - Si(3) - O(1)c	111.0(13)	110.3(6)	110.2
O(9) - Si(3) - O(1)c	108.0(8)	106.3(4)	105.2
O(2) - Si(4) - O(2)c	112.8(12)	108.3(6)	109.0
O(10) - Si(4) - O(2)c	107.4(7)	106.9(4)	105.6
**Average**	**109.5(1.8)**	**109.5(1.9)**	**109.4(2.7)**
**Min**	**107.2**	**106.3**	**105.2**
**Max**	**113.0**	**112.4**	**113.4**

Symmetry operations referred to the coordinates: c= x,y,1/2-z; f= 1/2-x,1/2-y,1-z

Table S4 Refined Si-O-Si angles for mordenite structure from dataset 1, 2 and the reference structure (Meier, 1961).

	**Dataset 1**	**Dataset 2**	**Meier (1961)**
Si(1) - O(1) - Si(3)	152.5(12)	150.2(6)	144.9
Si(3) - O(4) - Si(4)	170.9(14)	171.2(7)	166.4
Si(1) - O(7) - Si(1)a	147.0(14)	143.5(6)	136.5
Si(4) - O(10) - Si(4)b	142.4(15)	144.1(8)	148.0
Si(2) - O(2) - Si(4)	147.4(10)	145.3(5)	143.6
Si(2) - O(5) - Si(2)d	148.4(14)	147.7(7)	144.2
Si(2) - O(8) - Si(2)f	180.0	180.0	180.0
Si(1) - O(3) - Si(2)f	155.9(13)	157.0(6)	158.7
Si(1) - O(6) - Si(1)d	150.8(14)	150.2(6)	149.9
Si(3) - O(9) - Si(3)b	144.4(17)	144.2(9)	146.5
**Average**	**154.0(11.5)**	**153.3(12.0)**	**151.9(12.3)**
**Min**	**142.4**	**143.5**	**136.5**
**Max**	**180.0**	**180.0**	**180.0**

Symmetry operations referred to the coordinates: a= x,1-y,1-z; b= -x,y,1/2-z; d= x,y,3/2-z; f= 1/2-x,1/2-y,1-z

Table S5 Hirshfeld Rigid-Body test of the refined mordenite data from dataset 1 generated using PLATON (Spek, 2009).

**Atom(I)**	**Atom(J)**	**Obsd**	**I to J**	**J to I**	**Difference**	**Sqrt(Diff)**
Si(1)	O(1)	1.606(16)	0.059(4)	0.066(7)	0.007(9)	0.0837
Si(1)	O(3)	1.592(17)	0.046(4)	0.059(8)	0.013(9)	0.1140
Si(1)	O(6)	1.614(9)	0.078(4)	0.086(9)	0.008(10)	0.0894
Si(1)	O(7)	1.655(10)	0.057(4)	0.065(9)	0.008(10)	0.0894
Si(2)	O(2)	1.590(15)	0.044(4)	0.049(7)	0.005(9)	0.0707
Si(2)	O(5)	1.612(10)	0.071(4)	0.080(10)	0.009(11)	0.0949
Si(2)	O(8)	1.603(7)	0.073(4)	0.079(11)	0.006(12)	0.0775
Si(2)	O(3)f	1.617(16)	0.038(4)	0.062(8)	0.024(9)	0.1549
Si(3)	O(1)	1.590(16)	0.071(5)	0.080(7)	0.009(9)	0.0949
Si(3)	O(4)	1.628(19)	0.063(5)	0.073(10)	0.010(11)	0.1000
Si(3)	O(9)	1.598(12)	0.037(5)	0.042(11)	0.005(12)	0.0707
Si(4)	O(2)	1.601(16)	0.062(5)	0.071(7)	0.009(9)	0.0949
Si(4)	O(4)	1.632(19)	0.067(5)	0.073(10)	0.006(11)	0.0775
Si(4)	O(10)	1.630(11)	0.035(5)	0.035(11)	0.000(12)	0.0000

Sqrt(Sum(DelIJ**2)/Nrb) = 0.0087

Table S6 Hirshfeld Rigid-Body test of the refined mordenite data from dataset 2 generated using PLATON (Spek, 2009).

**Atom(I)**	**Atom(J)**	**Obsd**	**I to J**	**J to I**	**Difference**	**Sqrt(Diff)**
Si(1)	O(1)	1.610(8)	0.0391(18)	0.042(5)	0.003(5)	0.0548
Si(1)	O(3)	1.617(8)	0.0464(18)	0.044(4)	0.002(5)	0.0447
Si(1)	O(6)	1.612(4)	0.0359(18)	0.038(5)	0.002(5)	0.0447
Si(1)	O(7)	1.638(4)	0.0479(18)	0.053(5)	0.005(5)	0.0707
Si(2)	O(2)	1.612(7)	0.0350(18)	0.030(4)	0.005(4)	0.0707
Si(2)	O(5)	1.614(4)	0.0322(18)	0.033(6)	0.001(6)	0.0316
Si(2)	O(8)	1.604(3)	0.0514(18)	0.059(7)	0.008(7)	0.0894
Si(2)	O(3)f	1.598(7)	0.0439(18)	0.044(4)	0.000(5)	0.0000
Si(3)	O(1)	1.621(7)	0.035(2)	0.045(5)	0.010(5)	0.1000
Si(3)	O(4)	1.641(9)	0.048(2)	0.061(8)	0.013(8)	0.1140
Si(3)	O(9)	1.604(5)	0.035(2)	0.036(6)	0.001(6)	0.0316
Si(4)	O(2)	1.614(7)	0.033(2)	0.037(4)	0.004(5)	0.0632
Si(4)	O(4)	1.592(9)	0.052(2)	0.059(8)	0.007(8)	0.0837
Si(4)	O(10)	1.614(5)	0.032(2)	0.032(6)	0.000(6)	0.0000

Sqrt(Sum(DelIJ**2)/Nrb) = 0.0058
